# Exploring Older Adults’ Interest in Virtual Volunteering: Evidence from a Multi-Theoretical Model Combining TAM, Self-Efficacy, and Digital Divide Perspectives

**DOI:** 10.3390/bs15101340

**Published:** 2025-09-29

**Authors:** Longyu Sui, Jennifer A. Crittenden, Mark A. Hager

**Affiliations:** 1 School of Public Affairs, Arizona State University, Phoenix, AZ 85004, USA; 2 School of Social Work, University of Maine, Orono, ME 04469, USA; jennifer.crittenden@maine.edu; 3 School of Community Resources and Development, Arizona State University, Phoenix, AZ 85004, USA; mark.hager@asu.edu

**Keywords:** virtual volunteering, technology acceptance, digital divide, self-efficacy

## Abstract

The digital transformation of civic life has created new opportunities for older adults to engage in virtual volunteer activities. However, their participation still remains limited. This study investigates the factors that influence older adults’ interest in virtual volunteering. It integrated theoretical framework combining the Technology Acceptance Model (TAM), Self-Efficacy, and Digital Divide Theories to examine the drivers of virtual volunteerism interest among this target population. This study presents ordered logistic regression models with data on 814 adult volunteers in multiple imputation procedures. The final reduced model identifies two key predictors: a preference for virtual activities and interest in technology training, respectively, representing TAM and the Digital Divide Theory. While the self-efficacy-related variable showed statistical significance in earlier models, its explanatory power diminished when controlling for other factors. The findings indicate that older adults’ interest in virtual volunteering is primarily shaped by perceived usefulness of digital tools and their willingness to improve technical competence. This study confirms the relevance of the TAM and Digital Divide theories regarding virtual volunteerism. In practical terms, the findings indicate that program design should combine usability-focused platform features and targeted support that lower both technological and motivational barriers for older adults interested in virtual volunteering.

## 1. Introduction

### 1.1. Background

As global aging accelerates, promoting social participation among older adults has become more salient, especially in underserved regions (Koosha et al., 2025; Lim et al., 2025). Volunteering, to a deep extent, is widely recognized as a means of remaining active and maintaining well-being (He et al., 2025). The COVID-19 pandemic reshaped civic engagement by accelerating digitalization, making virtual volunteering a promising avenue for older adults to stay connected and involved (Wu & Zhu, 2025; Katey & Chivers, 2025). Virtual volunteering refers to the use of internet-based platforms that allow older adults to contribute their time, skills, or support to organizations or communities remotely—such as mentoring, administrative work, or online advocacy—without being physically present (Reuter et al., 2025). Despite its potential, actual engagement in virtual volunteerism remains low due to barriers such as digital illiteracy, limited access, low motivation, and anxiety, particularly among low-income and socially isolated individuals (Tomczyk & Kielar, 2025; Wang et al., 2025).

Contemporary definitions of “successful aging” now include not just health but digital competence and social connectedness (Kim & Freddolino, 2024). Evidence shows that social participation improves physical and cognitive health while reducing loneliness and isolation (Lim et al., 2025; Deslauriers & Levasseur, 2025; Ow et al., 2025). Still, a “policy–behavior gap” remains, as many older adults express interest in digital roles but face motivational, technical, and normative barriers (Kar et al., 2024; Katey & Chivers, 2025). The pandemic has further exposed urban–rural and intergenerational divides in digital readiness, underscoring the need to treat virtual volunteering as a behavioral process shaped by individual capacities and structural conditions (Lepore & McMullen, 2025; Cutamora et al., 2025). Research points to growing use of technology among older adults (AARP, 2025), coupled with the opportunities provided by the pandemic in shaping volunteering. However, participation in virtual volunteerism remains low at only 20% of individuals 55 and older reporting participating in some form of virtual or hybrid volunteering (Coleman & Crittenden, 2024).

### 1.2. Theoretical Framework

#### 1.2.1. Technology Acceptance Model (TAM)

The Technology Acceptance Model (TAM) was proposed by Davis (1989) and posits that an individual’s decision to adopt a particular technology is primarily influenced by two factors: perceived usefulness (i.e., whether the technology helps improve efficiency) and perceived ease of use (i.e., how easy the technology is to learn and operate). These two factors shape one’s intention together to use the technology and ultimately determine usage behavior.

A body of research confirms TAM’s explanatory power in the context of older adults’ digital technology adoption. In the realm of digital civic engagement, Reuter et al. (2025) found that older volunteers actively engage in website management, writing e-newsletters, and social media coordination—demonstrating a strong recognition of the utility of digital tools. In alignment with core TAM principles—particularly perceived usefulness and perceived ease of use—Wu and Zhu (2025) noted that digital tools like social media and health monitoring systems increased accessibility and service effectiveness. Similarly, Katey and Chivers (2025) emphasized how perceived ease of use and usefulness jointly shape older adults’ willingness to adopt tools such as Zoom and Skype, especially when these platforms are framed as intuitive and capable of reducing social isolation.

Prior studies have noted that difficulty understanding technical terminology and previous negative experiences with digital tools may cause anxiety and hinder perceived ease of use among older adults. Furthermore, the functionality and ease of use of ICT tools are pivotal factors in encouraging technology adoption among older adults, as highlighted by Tomczyk and Kielar (2025). Accordingly, instructional strategies should be designed to cultivate positive user perceptions during training sessions. Further, the digital divide remains a pressing concern. Many older people still experience exclusion because they have limited access to digital devices and lack sufficient technological proficiency. This underscores the structural barriers that limit the Technology Acceptance Model’s (TAM) applicability in contexts where people are digitally marginalized.

When individuals perceive that virtual engagement enables them to contribute meaningfully to their communities or enhances everyday functioning, their willingness to adopt digital tools correspondingly increases (Reuter et al., 2025; Wu & Zhu, 2025). Meanwhile, their willingness to learn new technologies or receive digital training is closely linked to their perceived ease of use. Factors such as technology-related anxiety, unfamiliar terminology, and low confidence in digital skills serve as practical barriers (Kar et al., 2024). Taken together, TAM provides a clear theoretical foundation for understanding whether older adults are willing to participate in virtual volunteering. Moreover, TAM provides a strong rationale for including explanatory variables such as “preference for virtual activities” and “willingness to learn new technologies.”

#### 1.2.2. Self-Efficacy in Social Cognitive Theory

A key component of social cognitive theory is self-efficacy, which refers to individuals’ beliefs in their ability to accomplish specific tasks. This has a significant influence on their motivation, emotional responses, and behavioral performance (Bandura et al., 1999; Deci et al., 1991). In the digital era, whether individuals believe they can competently use digital tools to manage new situations has become a crucial factor of their willingness to engage with technology. This makes the theory particularly relevant for understanding older adults’ readiness to adopt digital devices and participate in virtual volunteer activities.

Previous studies have demonstrated that self-efficacy is a key element in older adults’ ability to use technology and engage in social activities. For example, Lim et al. (2025) found that supportive interactions with volunteers helped older adults feel more capable and connected, which may enhance their self-efficacy. Drawing on case studies from the World Health Organization (WHO) and Japan, Koosha et al. (2025) stressed that volunteering can improve older adults’ awareness of their own abilities while also alleviating loneliness and depression.

In today’s digital world, low self-efficacy in older adults is frequently associated with memory or physical functioning declines, which result in fear of failure and reluctance to try new things (Katey & Chivers, 2025; Kar et al., 2024). Yet, research has indicated that older adults are highly motivated to acquire and experience a new sense of self-worth once they have established confidence (Kar et al., 2024). Tomczyk and Kielar (2025) also observed that hands-on demonstrations and practice-based instruction during Information and Communication Technology (ICT) training—referring to the use of digital tools such as computers, smartphones, and the internet to access, manage, and communicate information—significantly improved older adults’ confidence and willingness to engage proactively. Taken together, Self-Efficacy Theory provides a strong conceptual basis for explaining whether older adults are willing to participate in virtual volunteering.

#### 1.2.3. Digital Divide Theory

We adopt a standard, layered definition of the digital divide that distinguishes three dimensions: access, referring to the availability and affordability of devices, connectivity, and assistive technologies; skills, denoting the functional, informational, and problem-solving competencies required to use ICTs; and outcomes, which capture the realized benefits—and potential harms—accruing from use. In aging contexts, these dimensions intersect with place-based infrastructures and life-course resources, shaping who can convert access into meaningful participation. Within our framework, TAM reflects motivational appraisals of perceived usefulness and ease of use, self-efficacy represents perceived capability to act, and the digital divide situates these processes within broader structural constraints.

Digital Divide Theory emphasizes how disparities in access, skill levels, and technology usage outcomes lead to broader social inequalities (Katey & Chivers, 2025). The theory highlights that the digital divide is not merely an issue of internet access; it reflects deeper structural disparities rooted in income, education, and living conditions. These disparities tend to be particularly apparent among older adult populations (Gonzales et al., 2025; Wu & Zhu, 2025).

Wang et al. (2025) systematically categorize digital inequality into three dimensions: access gaps, support gaps, and usage gaps. They report that older adults with lower incomes are less likely to use the internet, and that digital exclusion is strongly associated with higher depression risk, especially among those in lower wealth brackets (Wang et al., 2025). Using data from Central and Eastern Europe, Tomczyk and Kielar (2025) found that over 30% of individuals aged 65 and above had never used the internet, highlighting the extent of digital exclusion in older populations. Urban planning also plays a crucial role in structural digital inequalities. Perez-Amado et al. (2025) note that many Canadian seniors live in low-density, car-dependent suburban areas lacking adequate digital infrastructure and training resources. They describe this situation as being “digitally excluded by design”—a form of structural digital inequality embedded in the spatial environment.

### 1.3. Gaps and Integrative Rationale

#### 1.3.1. Theoretical Modeling Rationale

Research documents how social participation enhances older citizens’ physical and psychological well-being. This offers a solid foundation for understanding potential interest in virtual volunteerism (Lim et al., 2025). However, population aging is inherently complex as it spans institutional, cultural, and technological boundaries. Thus, it demands a comprehensive theoretical framework (Koosha et al., 2025). In the period after the COVID-19 pandemic, as civic engagement increasingly shifted toward digital forms, studies have overlooked the unique motivations and capacities required for older adults’ digital participation (Reuter et al., 2025). Some studies, such as He et al. (2025), explore the interplay between psychological motivation and structural conditions in general volunteering contexts. However, few have extended this analysis to digital volunteerism, where the interaction between tech-related confidence, infrastructural access, and perceived usefulness may follow different patterns and present distinctive barriers. What’s missing are studies that focus specifically on how these factors shape interest in virtual volunteer engagement among older adults. Together, these theories provide a multidimensional framework linking psychological readiness, social influence, and structural inequality—laying the theoretical foundation for variable selection and empirical modeling in this study. At the micro level, disparities in digital literacy, social support, and engagement frequency persist (Deslauriers & Levasseur, 2025); at the meso level, living arrangements influence social capital and health outcomes (Zheng & Ni, 2025); and at the macro level, technology use and social participation have emerged as key themes in aging research (Kim & Freddolino, 2024). This study integrates the Digital Divide Theory, Self-Efficacy Theory, and Technology Acceptance Model (TAM) to clarify the structural, cognitive, and psychological factors that influence older adults’ interest in virtual volunteering. These theories are used in isolation, ignoring their potential for synergy at the micro, meso, and macro levels, despite being widely applied in research on technology use, behavioral beliefs, and digital inequality. Technology adoption is important because digital exclusion is connected with poorer mental health for older adults, especially those who with limited resources or family contact (Wang et al., 2025; Wu & Zhu, 2025). Technological engagement is shaped by perceived usefulness, self-efficacy, digital skills, and broader social environments (Katey & Chivers, 2025). However, volunteer programs often neglect these complexities, offering limited flexibility and support (Jung et al., 2025). A multi-theoretical approach is necessary to discuss the interplay of behavioral, cognitive, and structural factors.

#### 1.3.2. Summary of the Complementarity of the Three Theoretical Models

Three theoretical models—the Technology Acceptance Model (TAM), Self-Efficacy Theory, and Digital Divide Theory—are incorporated into the following empirical analysis. Together, they construct an explanatory chain that extends from individual perceptions to systemic constraints. These theories focus on four key elements: perceived ability, intention to act, actual access conditions, and willingness to use (Lim et al., 2025; Tomczyk & Kielar, 2025; Katey & Chivers, 2025).

The attitudes of older adults toward digital services and their willingness to use them are specifically explained by TAM. Their subjective belief in their own abilities is the main focus of the self-efficacy theory. Institutional and structural factors restrict access to technology, as identified by the Digital Divide Theory (Reuter et al., 2025; Wang et al., 2025; Gonzales et al., 2025). The combination of these models forms a complementary mechanism that captures both the psychological motivations and behavioral processes of older adults’ adoption of technology. While these theories are widely applied in research on digital inequality, behavioral beliefs, and technology use, they are frequently employed in isolation, ignoring their potential for synergy at the micro, meso, and macro levels. TAM highlights perceived utility and usability as important factors that influence the adoption of technology (McGinty et al., 2024; Li et al., 2025; Jia et al., 2015), but it does not adequately account for social context and perceived behavioral control, particularly among older adults (Lee et al., 2025). For this group, perceiving a technology as “easy to use” is insufficient without the belief in their ability to use it effectively—making Self-Efficacy Theory essential. Focusing on motivational beliefs and perceived competence, Self-Efficacy Theory has been shown to explain older adults’ engagement with digital technologies (Warner et al., 2011; Müller et al., 2014), particularly through internet self-efficacy (Lam & Lee, 2005; Jokisch et al., 2023). Nonetheless, behavioral variations among individuals with similar self-perceptions highlight the drawbacks of relying solely on structural constraints to explain results. In order to address these gaps, the Digital Divide Theory identifies institutional and resource-based obstacles to digital participation including skills, usage outcomes, and access (Mukherjee, 2011; Jones et al., 2015; Lozoya et al., 2022; Quan-Haase et al., 2018; An et al., 2024).

### 1.4. Theoretical Framework and Study Aim

**Figure 1:**
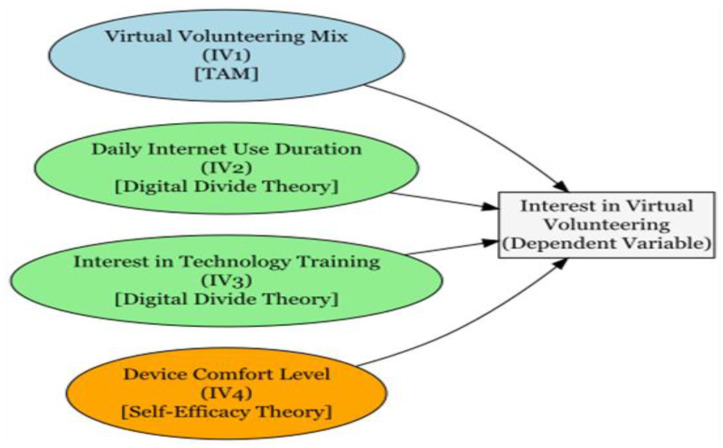
Conceptual framework linking TAM, Digital Divide Theory, and Self-Efficacy Theory to older adults’ interest in virtual volunteering.

This study integrates three behavioral theories—Technology Acceptance Model (TAM), Self-Efficacy Theory, and Digital Divide Theory—within the broader resource-based framework proposed by He et al. (2025), in order to systematically explain older adults’ involvement in virtual volunteering. TAM emphasizes perceived utility and ease of use as key factors influencing the adoption of technology (Davis, 1989; Kar et al., 2024). Self-Efficacy Theory focuses on individuals’ confidence in mastering digital tools (Katey & Chivers, 2025; Gonzales et al., 2025). Digital Divide Theory captures structural barriers related to access, skills, and inequality (Tomczyk & Kielar, 2025; Wang et al., 2025). Many studies follow the logic of these models, even if not all adopt them explicitly. For instance, Wu and Zhu (2025) highlight the role of infrastructure and support in digital adaptation. Katey and Chivers (2025) bridge perspectives from TAM and Digital Divide Theory. Meanwhile, Wirth et al. (2025) trace how internalized norms shape the formation of behavioral intentions. Reuter et al. (2025) further support the integration of motivation, skills, and digital behavior via self-efficacy. Together, these theories provide a multidimensional framework linking psychological readiness, social influence, and structural inequality—laying the theoretical foundation for variable selection and empirical modeling in this study. Figure 1 illustrates this integrated framework, mapping how the three theories jointly inform our explanation of older adults’ interest in virtual volunteering.

## 2. Materials and Methods

### 2.1. Data Source and Sample

Our study draws on surveys collected from 814 older adult volunteers participating in AmeriCorps Seniors programs, including Senior Companion, Foster Grandparent, RSVP, and the Senior Demonstration Program. The Senior Companion Program engages older adults in volunteer assignments where they provide companionship to other older adults who need assistance with daily tasks. The Foster Grandparent Program matches older adult volunteers with youth to provide intergenerational mentorship and support that helps to them build academic and emotional well-being. Both the RSVP and Senior Demonstration Program deploy older adult volunteers locally across a variety of volunteer opportunities based on local needs, AmeriCorps funding priorities, and volunteer interests (AmeriCorps, n.d.). Survey data were collected under a federal contract to examine virtual volunteering among older adults and the associated challenges and opportunities with virtual volunteering practices. Data were collected from a sample of programs from 25 states across the United States. The survey period ran from January through April 2025 via an online survey administered via the Qualtrics survey platform. The survey included questions about program volunteer experiences with virtual and phone-based volunteering. Questions were a mix of yes/no, multiple choice, open-ended, and 5-point Likert scale questions. Questions were developed based on literature review and existing measures. Of the 814 surveyed AmeriCorps Seniors participants across 25 U.S. states, our analytic sample comprises 699 cases with non-missing values on the dependent variable. For independent variables with missingness, we applied multiple imputations (chained equations) and estimated all models on the imputed datasets.

### 2.2. Variables and Methods

To connect the theoretical framework with the empirical strategy, this section offers an integrated conceptual mapping of the study’s dependent and independent variables, anchored in the Technology Acceptance Model (TAM), Self-Efficacy Theory, and Digital Divide Theory. Rather than rigidly aligning each variable with a single framework, this study adopts a cross-theoretical perspective to better capture the multifaceted nature of older adults’ engagement with digital volunteerism. The selected theoretical models serve not only to explain behavioral patterns but also to structure the empirical logic behind the ordered logistic regression models.

We employed a stepwise modeling strategy to assess the incremental contribution of each theoretical framework. Model 0 included only control variables. Models 1–3 added predictors from each framework in turn—TAM, digital divide, and self-efficacy together with controls. Model 4 combined all theoretical predictors with controls. Finally, Model 5 retained a subset of theoretically relevant and statistically significant predictors from Model 4, along with selected controls. This approach allows us to compare the explanatory contributions of the frameworks individually and in combination, while maintaining theoretical consistency and interpretability. As part of this process, we also explored alternative specifications; although some produced higher in-sample fit, they were less aligned with our theory-driven rationale and raised concerns about overfitting. We therefore prioritized theoretical coherence over maximizing fit in the final specification.

The dependent variable in this study is participants’ interest in increasing virtual volunteering activities, measured by a survey item asking respondents how interested they are in expanding their virtual volunteer roles. Response options range from “Not at all interested” to “Extremely interested,” on a five-point ordinal scale.

The first independent variable, virtual volunteerism participation, captures respondents’ existing engagement in virtual formats by asking them to report the current proportion of their in-person versus virtual volunteering activities. Responses reflect a continuum from fully in-person to fully virtual, and are coded in ascending order from 1 to 5 to represent increasing digital participation. This variable serves as a behavioral proxy for TAM (through reinforcement of perceived usefulness). This variable replicates the one used in the 2023 Civic Engagement and Volunteering Supplement of the Census Current Population Survey (U.S. Census Bureau, 2024).

The second independent variable assesses daily internet exposure through a question about how much time respondents typically spend online each day. Responses were provided in categorical time ranges, which were then converted into approximate numeric values to better capture the continuous nature of digital engagement: “No time” was coded as 0 h; “Less than an hour” as 0.5; “1–2 h” as 1.5; “2–5 h” as 2.5; “5–10 h” as 7.5; and “10 or more hours” as 15. This recoding allows for a more precise representation of individuals’ habitual online activity. The variable serves as a proxy for digital familiarity and frequency of use, and is conceptually aligned with the first-level digital divide, which emphasizes unequal access to internet infrastructure, bandwidth, and usage opportunities across demographic groups.

The third independent variable reflects interest in acquiring new technology skills, measured by a question asking respondents how interested they are in participating in digital training programs. Responses are rated on a five-point scale from “not at all interested” to “extremely interested.” This variable reflects individuals’ motivation to address their gaps in digital skills. It falls within the concept of the “digital divide theory,” which focuses on differences in individuals’ ability and willingness to use digital technology. As such, the variable serves as an important indicator of respondents’ orientation toward digital capacity and their potential to overcome existing barriers to meaningful technology engagement.

The fourth independent variable, Overall Device Comfort, is derived by averaging responses to three items that assess comfort with using a smartphone, tablet, and laptop or desktop computer. Each item asks, “How comfortable are you with using [device]?”, with response options ranging from “N/A = I have never used this device” to “5 = Extremely comfortable.” The composite index, grounded in Self-Efficacy Theory which emphasizes individuals’ belief in their ability to perform specific tasks, captures cross-device technological confidence. In this context, the measure reflects self-perceived competence with common digital tools, which may shape respondents’ willingness to engage in technology-mediated civic activities. We operationalize self-efficacy using a composite “device comfort” index (averaged from three items).

Although these variables function as proxy measures, they collectively reflect a developmental trajectory of digital participation—beginning with access to online technologies, followed by behavioral engagement, motivational orientation toward skill acquisition, and culminating in self-perceived digital competence. This sequence aligns with the concept of a “structural digital differentiation chain,” which captures the layered nature of digital inequality shaped by factors such as education, income, and social support (Perez-Amado et al., 2025; Wu & Zhu, 2025; Zheng & Ni, 2025). Control variables—such as educational attainment, volunteer years, and age—further account for structural and relational forms of capital relevant to digital inclusion (Wang et al., 2025; Zheng & Ni, 2025). By situating these variables within a multi-theoretical framework, this study establishes a clear conceptual foundation for empirical analysis. The integration of TAM, Self-Efficacy Theory, and Digital Divide Theory enables a nuanced understanding of the barriers and enablers shaping older adults’ digital engagement in virtual volunteerism.

### 2.3. Analytical Strategy

This study develops an analytical framework based on older adults’ interest in virtual volunteering. Key variables were selected grounded in established theoretical frameworks and empirical evidence to ensure both rigor and explanatory power. Drawing on Lim et al.’s (2025) concept of reciprocal participation—where older adults act as both service recipients and contributors—the variable representing preference for virtual formats captures this dual-role logic. The core variables identified through Lasso regression include virtual volunteering mix, daily internet use duration, interest in technology training, and devices comfort level.

Each of these maps onto structural engagement, motivational intention, and perceived capability. Each also aligns with TAM, self-efficacy, and digital divide perspectives (Koosha et al., 2025; Reuter et al., 2025). This modeling strategy—Multiple imputation, Lasso selection, then Ordered logit analysis—follows Cheung’s (2024) “cluster first, explain later” approach, which effectively captures specialty in older adults’ social engagement. Additional theoretical grounding comes from studies linking social networks to digital competence (Deslauriers & Levasseur, 2025) and highlighting the interaction between residential context and social capital (Zheng & Ni, 2025), which informs the inclusion of moderating variables such as social support. Control variables were selected based on prior findings that demonstrate their influence on older adults’ willingness to increase engagement in virtual volunteering (He et al., 2025; Wang et al., 2025; Tomczyk & Kielar, 2025). These factors enhance model robustness by accounting for individual-level resource conditions. In sum, the selected variables reflect behavioral, cognitive, and structural dimensions of digital volunteering among older adults. This theory-informed selection provides a solid foundation for the subsequent ordered logit modeling.

## 3. Results

Table 1 presents descriptive statistics for the study sample, which consists of 699 older adult volunteers aged 55 and above. Of the 814 total respondents, 699 provided valid responses to the dependent variable used in this study. The remaining 115 cases were excluded due to missing outcome data. Among the 699, some independent and control variables had missing values, which were addressed using multiple imputation. The final analytic sample therefore includes 699 respondents with complete outcome data and imputed values for selected predictors. The average age of respondents was 72.1 years (SD = 6.8), with a range from 53 to 94 years. On average, participants reported 7.7 years of volunteering experience (SD = 6.5) and 16.4 years of formal education (SD = 3.2), indicating a relatively well-educated and experienced cohort. Regarding key independent variables, most respondents primarily engaged in in-person volunteering, with an average volunteering modality score of 1.5 (range = 1–5; higher values reflect greater involvement in virtual formats). Participants reported an average of 2.1 h of daily internet use (SD = 1.7), with responses ranging from no use to 15 h per day.

The mean score for interest in digital skills training was 2.7 (SD = 1.1), suggesting a moderate level of motivation to improve technological competencies. Technology self-efficacy was assessed using a device comfort index, calculated as the average of comfort ratings for using a smartphone, tablet, and computer. The mean index score was 3.8 (SD = 0.9, out of a possible 5), reflecting a moderate level of confidence across multiple digital platforms. Notably, due to some participants having never used certain devices, the valid sample size for this variable was 691. The dependent variable—interest in increasing virtual volunteering participation—had a mean score of 2.2 (SD = 1.1), indicating a generally low to moderate level of enthusiasm for expanding engagement in digital forms of volunteerism.

This study sequentially built six ordered logistic regression models (M0–M5) after multiple imputation in order to examine the main factors influencing older adults’ interest in taking part in virtual volunteer activities. The dependent variable is participants’ interest in increasing virtual volunteering activities. The Digital Divide Theory, Self-Efficacy Theory, and Technology Acceptance Model (TAM) were used to guide the selection of independent variables. Four control variables were taken into account by models M0 through M4: education level, years of volunteer experience, age, and gender. Three variables remained in the final reduced model (M5): C3 (Years of Volunteer Experience), C4 (Education Level), IV3 (Interest in Technology Training), and IV1 (Virtual Volunteering Mix).

Table 2 presents the main logistic regression results for all models. First, IV1, a key variable from the Technology Acceptance Model (TAM) that reflects the preference between in-person and online volunteering modes, demonstrated stable and significant predictive power across all models. In Model 1, its regression coefficient was β = 0.535 (*p* < 0.001), and it remained significant in the integrated Model 4 (β = 0.462, *p* < 0.001) and Model 5 (β = 0.454, *p* < 0.001). This pattern suggests that a stronger preference for online volunteering corresponds with a greater willingness among older adults to engage in virtual volunteer activities. This finding supports the view of Jokisch et al. (2023), who argued that perceived usefulness promotes older adults’ adoption of virtual volunteering platforms. Similarly, Reuter et al. (2025) noted that older volunteers who involved in virtual tasks often expressed strong appreciation for the practical value of technology. Wu and Zhu (2025) further emphasized that “perceived usefulness” and “perceived ease of use” jointly form the basis of older adults’ intentions to adopt technology, especially when tools like Zoom or health-monitoring apps enhance accessibility and convenience.

Next, the digital divide-related variables showed varied performance across models. IV3, which captures interest in technology training, was statistically significant in Model 2 (β = 0.941, *p* < 0.001) and remained robust in Model 4 (β = 0.946, *p* < 0.001) and Model 5 (β = 0.944, *p* < 0.001). This further highlights the essential role of “learning motivation” in driving older adults’ engagement in virtual volunteerism (Müller et al., 2014; Jokisch et al., 2023). It also echoes a central proposition of self-efficacy theory which is that when individuals’ perceived abilities are enhanced, their willingness to participate increases accordingly. In contrast, IV2 (Daily Internet Use Duration) exhibited a moderate level of significance in Model 2 (β = 0.121, *p* = 0.005). However, its predictive power declined in the full models, though it remained marginally significant in Model 5 (*p* = 0.037). This suggests that while routine internet use may contribute to greater digital familiarity, its impact is more limited compared to targeted learning motivation. Overall, IV3 stands out as the strongest explanatory variable among digital divide indicators. As Mukherjee (2011) noted, older adults’ engagement in virtual services depends not only on access to technology but also on frequent exposure and sustained motivation to learn. Lozoya et al. (2022) similarly emphasized that the use of ICT for communication and learning is positively associated with self-efficacy in older adults. Taken together, these studies suggest that regular interaction with online learning tools or social media can boost older adults’ confidence and increase their willingness to participate in virtual volunteering.

Finally, IV4 (Device Comfort Level), which is grounded in self-efficacy theory, was statistically significant in Model 2 (β = 0.298, *p* = 0.001), but lost its significance in Model 4 (*p* = 0.061), and was excluded in the final Model 5, indicating its relatively weak explanatory power. This suggests that its standalone effect may be mediated or moderated by other variables. This finding supports the argument by Warner et al. (2011), who emphasized that mastery experience and vicarious experience are more critical for the development of self-efficacy than mere comfort with operating devices. IV4 is more likely to represent one specific dimension of the broader self-efficacy mechanism in digital environments—namely, operational confidence—rather than being a decisive factor on its own.

In the early models, two control variables showed statistical significance. C3 (Years of Volunteering) was significant in Model 1 (β = −0.025, *p* = 0.029), and C4 (Education Level) reached significance in Model 2 (β = 0.052, *p* = 0.022). Although their significance weakened in the subsequent models, the final model (M5) retained both variables. In M5, the regression coefficient of C3 was −0.022 (*p* = 0.050), remaining statistically significant, while the coefficient of C4 was 0.043 (*p* = 0.058), marginally significant. This indicates that older adults with longer volunteering experience may be less inclined to participate in virtual volunteering, whereas those with higher educational attainment may be more interested. However, their effects may have been partially absorbed after the inclusion of main effect variables such as IV1 and IV3.

To sum up, the final reduced model (M5) reveals that the two most powerful predictors of older adults’ interest in virtual volunteering are IV1 (Virtual Volunteering Mix) from the Technology Acceptance Model and IV3 (Interest in Technology Training) from the Digital Divide Theory. These two variables correspond to the theoretical pathways of “motivation to acquire capability” and “perceived usefulness,” respectively. Both remained highly significant even after controlling for other variables, indicating that older adults’ willingness to engage in virtual volunteer activities is primarily driven by their proactive attitude toward skill acquisition and their recognition of the value of technology. Although self-efficacy-related variables such as IV4 lost significance in the final model, their conceptual importance may have been indirectly captured through the digital divide constructs. Overall, the study confirms the relevance and complementarity of the Technology Acceptance Model and Digital Divide Theory in explaining older adults’ motivations for virtual volunteerism and provides strong theoretical support for the design of future interventions and policymaking.

## 4. Discussion

This study employed an integrated analytical framework that combines the Technology Acceptance Model (TAM), digital divide theory, and self-efficacy theory to systematically examine the key mechanisms that influence older adults’ willingness to engage in virtual volunteering. Using a series of ordered logistic regression models, the final streamlined model (M5) identified two significant explanatory variables—Virtual Volunteering Mix (IV1) and Interest in Technology Training (IV3)—along with one control variable, Education Level (C4). These findings suggest that behavioral familiarity with digital forms and a tendency toward motivation–capability development are the two main explanatory pathways that drive virtual volunteer engagement.

To begin with, IV1 remained consistently significant across Models 1, 4, and 5, demonstrating the central role of actual digital behavior in shaping older adults’ future willingness to participate. The effect of IV1 was robust across models (β = 0.476, *p* < 0.001 in M5). This indicates that individuals who are currently more involved in virtual volunteering are more likely to express continued or increased interest in digital participation. Rather than reflecting cognitive judgments such as perceived usefulness alone, this pattern underscores the importance of behavioral routines and prior exposure in fostering digital engagement tendencies. This interpretation aligns with existing literature on the self-reinforcing mechanism of digital behavior. For example, Cheung (2024) found that older adults who adapted to virtual interaction during the COVID-19 pandemic tended to maintain those digital habits even after restrictions were lifted. Similarly, Katey and Chivers (2025) pointed out that older individuals who regularly maintained community connections via digital platforms experienced a significant increase in digital confidence and were more likely to continue participating in virtual activities. Collectively, these studies suggest that individuals already integrated into hybrid or fully virtual volunteering environments face fewer psychological and technological barriers, making it easier for them to sustain online engagement.

Secondly, IV3 (Interest in Technology Training) remained consistently significant across multiple models (e.g., β = 0.937, *p* < 0.001 in M5), indicating that training motivation is a critical factor in promoting digital inclusion—particularly when older adults view learning as a pathway to achieving personal goals. Cheung (2024) notes that self-initiated training, combined with frequent technology use, enhances older adults’ online and social engagement, revealing a positive association between cognitive readiness and opportunities for digital participation. Kar et al. (2024) found that when training content is closely aligned with real-life scenarios, older adults’ motivation to participate increases significantly, further supporting the explanatory power of IV3. This variable also highlights the second-level digital divide—not merely access, but also the willingness and capability to learn and cross the threshold (Koosha et al., 2025). Reuter et al. (2025) emphasize that digital citizenship must be built on a continuous process of learning–adaptation–participation. IV3 also reflects variation in types of digital participation among older adults; Choudrie et al. (2022) emphasize that targeted training helps alleviate structural barriers faced by older adults from minority backgrounds, while Jokisch et al. (2023) report that learning opportunities in ICT-based volunteer services significantly enhance both access and sustainability among older adults. Overall, IV3 exhibits high stability across related models, clearly reflecting its role as a representative indicator of the “motivation gap” within the digital divide framework.

In contrast, even though IV2 (Daily Internet Use Duration) showed statistical significance in Model 2, IV2 was not included in the final model. This result underscores a distinction between passive exposure and active participation. While internet exposure is a necessary foundation, it does not automatically translate into deeper forms of digital civic engagement such as virtual volunteering. Relatedly, Gonzales et al. (2025) emphasize that older adults with higher educational attainment are more likely to possess cognitive resources, openness to technology, and greater social capital, all of which enhance willingness and capacity to participate in virtual volunteering. However, as Wang et al. (2025) argue, frequent internet use does not necessarily indicate digital readiness, since many older adults engage in online activities passively or without clear goals. Likewise, Jung et al. (2025) underscore heterogeneity in older adults’ motivations for technology use, suggesting that usage frequency alone is an inadequate indicator of digital engagement or preparedness. Here too, volunteer programs should justify virtual volunteer assignments by helping volunteers understand the linkage between internet use and the outcomes that result for program beneficiaries—for example, showing how connectivity supports building relationships with youth mentees.

It is also worth noting that the number of years in volunteering (C3) reached statistical significance in both Model 1 (*p* = 0.029) and Model 5 (*p* = 0.050), suggesting that—regardless of modality—prior volunteer experience may positively influence willingness to engage in virtual volunteering. This result may reflect a stronger sense of civic responsibility or service identity, making individuals more adaptable to new forms of participation. In contrast, educational level (C4) was only significant in Model 2 (*p* = 0.022) and became marginally significant in Model 5 (*p* = 0.058), indicating a relatively unstable influence. Although educational attainment is typically regarded as relevant to digital readiness and civic engagement, its explanatory power may be overshadowed by more proximal variables such as interest in technology training or prior behavioral experience. Nevertheless, existing research continues to emphasize its theoretical importance. For example, Gonzales et al. (2025) argue that education enhances individuals’ cognitive adaptability and openness to new technologies. In this vein, Koosha et al. (2025) and Tyler et al. (2020) link education to lifelong learning capacity and civic motivation.

While Model 4 exhibited the best overall fit, the final model (M5) was selected for its balanced combination of theoretical clarity and statistical robustness. It retains variables that remained consistently significant across models, offering a more parsimonious specification without sacrificing theoretical integrity.

Taken together, the final model (M5) confirms the explanatory power of the integrated analytical framework. After controlling for a range of demographic and behavioral variables, both Virtual Volunteering Mix (IV1) and Interest in Technology Training (IV3) remain statistically significant. This indicates that willingness to engage in virtual volunteering is primarily driven by actual behavioral experience, intrinsic motivation, and civic engagement history, rather than by mere access to technology or demographic characteristics. In comparison, although IV4 (Device Comfort Level) did not reach statistical significance in M5, its presence in earlier models suggests the possibility of indirect pathways—particularly through self-efficacy and learning motivation.

The self-efficacy proxy did not achieve statistical significance in Model 4 and, accordingly, was not retained in Model 5. We caution against interpreting this as evidence of theoretical irrelevance. First, our measure operationalizes self-efficacy via a composite “device comfort” index, which may not fully capture the multidimensional psychological construct. Second, characteristics of the sampling frame—AmeriCorps Seniors participants who may already possess relatively high baseline confidence—could compress variance. As an additional robustness check, we tested multicollinearity using variance inflation factors (VIFs); all values were within acceptable thresholds, indicating that collinearity is unlikely to account for the non-significance. We offer this clarification to highlight the continued theoretical importance of self-efficacy for future research and measurement.

To translate these findings into practice, platforms and programs should prioritize ease of use and low-friction engagement pathways for older adults. Concretely: (a) implement large fonts, high-contrast themes, plain-language prompts, and consistent navigation; (b) minimize steps to start (e.g., password-less or assisted login, single-task screens, and clear progress cues); (c) provide step-by-step onboarding with short, task-specific tutorials and a demo/sandbox mode; (d) offer multimodal support (phone line, live chat, or local partner help desks) and low-bandwidth compatibility; and (e) align tasks with volunteers’ existing skills and civic identities (e.g., flexible micro-tasks, recognition of contributions, and role matching). These design and program features are likely to activate intrinsic motivation while reducing technological and motivational barriers to sustained virtual volunteering.

Zheng and Ni (2025) found that frequent visits from adult children mitigate depressive symptoms among older parents, particularly in rural areas where community services are limited; conversely, urban environments can mask resource gaps behind high population density. Wang et al. (2025) systematically categorize digital inequality into access, skills, and outcomes, showing that digital exclusion is strongly associated with higher depression risk, especially among those in lower wealth brackets. Urban planning also plays a crucial role in structural digital inequality through the uneven distribution of broadband, devices, and age-friendly learning resources. They describe this situation as being “digitally excluded by design”—a form of structural digital inequality embedded in the spatial environment.

However, this study is subject to several limitations. First, the cross-sectional nature of the data limits the ability to draw causal inferences between predictors and interest in virtual volunteering. Second, the use of secondary data constrains the selection and measurement of variables to those already available. For example, we operationalize self-efficacy using a composite index. As a proxy, this measure may not fully capture the multidimensional psychological construct of self-efficacy, potentially missing subjective nuances. However, to the authors’ knowledge, no existing large-scale datasets currently allow for this type of inquiry into virtual volunteering participation and the triangulation of behavioral theory, underscoring the value of this exploratory effort. Third, related to the cross-sectional design, we cannot capture trajectories of change in motivation and digital engagement over time. Because the sampling frame consists of AmeriCorps program participants, generalizability beyond similar older-adult volunteer populations should be interpreted with caution. Likewise, the sample may be biased toward older adults who are already embedded in volunteering networks limiting generalizability to the broader older adult population and should be interpreted accordingly.

## 5. Conclusions

When individuals perceive that virtual engagement enables them to contribute meaningfully, sustain social roles, and maintain identity in later life, they are more likely to develop intrinsic motivation to participate. Their willingness to learn new technologies or undertake digital training likewise reflects a subjective judgment about the feasibility and value of engagement. Although our analysis is quantitative, older adults’ lived experience, agency, and later-life identity are central to digital participation. Incorporating qualitative approaches would enrich interpretations of motivations, barriers, and the social meanings attached to virtual volunteering.

This study finds that willingness to engage in virtual volunteering is shaped by perceived usefulness and motivation to receive technology training—mechanisms at the core of the Technology Acceptance Model (TAM) and Digital Divide Theory. Although variables derived from Self-Efficacy Theory did not exhibit consistent statistical significance, their theoretical and contextual relevance remains important. Consequently, policies should move beyond basic technological access to emphasize motivation and capacity building. In practical terms, this entails providing socially meaningful digital platforms, designing training that is grounded in everyday life, and fostering supportive learning environments to increase older adults’ interest and engagement in virtual volunteering. Although the self-efficacy proxy was not statistically significant in our final specification, we interpret this as a measurement and sampling constraint rather than a challenge to its theoretical relevance, underscoring the need for more direct, multidimensional measures in future work. Future research should also incorporate longitudinal and qualitative designs to trace developmental pathways and contextual meanings.

## Figures and Tables

**Table 1:** Descriptive Statistics for Key Variables.

Variables/Numbers	n	Mean	SD	Min	Max
Dependent Variable	699	2.15	1.10	1	5
Age(C1)	699	72.13	6.76	53	94
Years of Volunteering(C3)	699	7.68	6.46	0.5	25
Education Level (C4)	699	16.4	3.19	8	21
Virtual Volunteering Mix (IV1)	699	1.47	0.84	1	5
Daily Internet Use Duration (IV2)	699	2.12	1.73	0	15
Interest in Technology Training (IV3)	699	2.71	1.12	1	5
Device Comfort Level (IV4)	691	3.8	0.85	1	5

**Table 2:** Ordered Logistic Regression Results for Models M0–M5.

Variable/Model	M0	M1	M2	M3	M4	M5
Age estimate (C1)	−0.015	−0.016	−0.020	−0.008	−0.017	
Gender estimate (C2)	0.077	0.098	0.095	0.041	0.107	
Years of Volunteering estimate (C3)	−0.018	−0.025	−0.008	−0.017	−0.014	−0.022
Education Level estimate (C4)	0.041	0.021	0.052	0.032	0.037	0.043
Virtual Volunteering Mix estimate (IV1)		0.535			0.462	0.476
Daily Internet Use Duration estimate (IV2)			0.121		0.091	
Interest in Technology Training estimate (IV3)			0.941		0.946	0.937
Device Comfort Level estimate (IV4)				0.298	0.175	
Age *p*-value (C1)	0.175	0.157	0.079	0.502	0.163	
Gender *p*-value (C2)	0.642	0.556	0.578	0.808	0.54	
Years of Volunteering *p*-value (C3)	0.125	0.029 *	0.495	0.139	0.269	0.050 *
Education Level *p*-value (C4)	0.065	0.352	0.022 *	0.150	0.118	0.058
Virtual Volunteering Mix (IV1)		0.000 ***			0.000 ***	0.000 ***
Daily Internet Use Duration *p*-value (IV2)			0.005 **		0.037 *	
Interest in Technology Training *p*-value (IV3)			0.000 ***		0.000 ***	0.000 ***
Device Comfort Level *p*-value (IV4)				0.001 ***	0.061	
Pseudo R^2^	0.005	0.025	0.109	0.012	0.131	0.118
N	699	699	699	691	691	699
AIC	1931	1896	1735	1895	1675	1714
BIC	1967	1937	1780	1935	1730	1750

Note: * *p* < 0.05, ** *p* < 0.01, *** *p* < 0.001. Coefficient estimates are shown with significance levels indicated by asterisks. *p*-values are rounded to three decimal places.

## Data Availability

The dataset used in this analysis is available by request from the second author, J.C.

## Abbreviations

The following abbreviations are used in this manuscript:

ICT	Information and Communication Technology
TAM	Technology Acceptance Model
